# Supplementary material for: Relationship between customer knowledge management and the value co-creation of fitness application customers: Mediating role of flow experience
Source: PLoS One. 2024 Oct 29;19(10):e0311988. doi: 10.1371/journal.pone.0311988 (PMC11521290; doi:10.1371/journal.pone.0311988)
Supplement: S1 Appendix — (PDF) [file pone.0311988.s002.pdf]

Questionnaire

Dear Mr / Ms :

Hello ! We are the students of Wuhan Institute of Physical Education. We are studying the user behavior of sports fitness APP, and hope that you can help complete this questionnaire. The data collected in this questionnaire is only used for academic research. Your answers will be completely confidential. The content you feedback is very important for this study. Thank you for your support. I wish you good health and all the best !

Answer description :

This questionnaire takes customers who have participated in sports and fitness APP as the survey object (such as keep, yuedong, gudong, etc.). If ' participated ', please continue to fill out this questionnaire ; " Not involved " Please ignore this survey. Once again sincerely thank you for your cooperation, I wish you good health, all the best ! In addition, the word " enterprise " in the following table refers to sports and fitness APP.

The first part :

Tick the options that you think are most suitable (completely disagree=1, disagree=2, slightly disagree=3, uncertain=4, slightly agree=5, agree=6, completely agree=7) based on the degree of conformity between the actual feelings you use and the following statements.

Variable/items	1	2	3	4	5	6	7
CUSTOMER KNOWLEDGE MANAGEMENT							
**THE APP ASKS ME ABOUT WHAT ATHLETIC ACTIVITIES I AM INTERESTED IN AND HOW HIGHLY I RATE THE QUALITY OF SERVICE.**							
**THE APP UNDERSTANDS MY NEEDS AND SOLVES MY PROBLEMS IN A TIMELY MANNER.**							
**THE APP HAS A GOOD GRASP OF MY FITNESS PREFERENCES.**							
**THE APP PROVIDES ME WITH UPDATES AND INFORMATION ON NEW PRODUCTS.**							
**THE APP OFFERS SUGGESTIONS TO HELP ME MAKE BETTER CONSUMPTION DECISIONS.**							
PERCEIVED ENJOYMENT							
**IT'S INTERESTING TO USE THE APP.**							
**IT'S EXCITING TO USE THE APP.**							
**IT'S PLEASANT TO USE THE APP.**							
**IT'S ENJOYABLE TO USE THE APP.**							
ATTENTION FOCUS							
**I AM HIGHLY ATTRACTED TO THE APP'S PRODUCTS AND SERVICES.**							
**I AM IN A STATE OF DEEP FOCUS WHEN USING THE APP.**							
**I AM IN A STATE OF DEEP FOCUS WHEN USING THE APP'S PRODUCTS AND SERVICES.**							
**I GIVE MY ALL TO THE ATHLETIC ACTIVITIES ASSOCIATED WITH THE APP.**							
CUSTOMER PARTICIPATION BEHAVIOR							
INFORMATION SEEKING							
**I ASK OTHERS ABOUT ATHLETIC PRODUCTS AND SERVICES.**							
**I HAVE SEARCHED FOR LOCATION INFORMATION ABOUT SPORTS PRODUCTS AND SERVICES**							
**I HAVE OBSERVED HOW OTHER PEOPLE USE FITNESS APPS.**							
INFORMATION SHARING							
**I HAVE CLEARLY EXPRESSED MY NEEDS WITH REGARD TO THE PRODUCTS OR SERVICES PROVIDED BY THE COMPANY.**							
**I HAVE PROVIDED USEFUL INFORMATION TO HELP THE COMPANY INNOVATE IN THEIR PRODUCTS AND SERVICES.**							
**I HAVE OFFERED FEEDBACK ON HOW THE COMPANY CAN PROVIDE SERVICES IN A TIMELY MANNER.**							
**I ANSWER ALL QUESTIONS POSED TO ME ABOUT SPORTS PRODUCTS AND SERVICES.**							
RESPONSIBLE BEHAVIOR							
**I HAVE COMPLETED ALL THE STEPS REQUIRED BY THE**							

APP.

I HAVE UNDERTAKEN ALL ACTIONS REQUIRED BY THE APP.

I HAVE FULFILLED ALL TASKS REQUIRED BY THE APP.

I HAVE COMPLIED WITH ALL INSTRUCTIONS GIVEN BY THE APP.

PERSONAL INTERACTION

I AM FRIENDLY TO CUSTOMER SERVICE STAFF ONLINE AND OFFLINE.

I AM KIND TO CUSTOMER SERVICE STAFF ONLINE AND OFFLINE.

I AM POLITE TO CUSTOMER SERVICE STAFF ONLINE AND OFFLINE.

I AM COURTEOUS TO CUSTOMER SERVICE STAFF ONLINE AND OFFLINE.

I AM FRIENDLY TO CUSTOMER SERVICE STAFF ONLINE AND OFFLINE.

CUSTOMER CITIZENSHIP BEHAVIOR

FEEDBACK

I LET THE COMPANY KNOW WHEN I HAVE ANY FEEDBACK.

I AM WILLING TO COMMENT ON CONTENT ON THE APP WHENEVER I ENCOUNTER IT.

I LET THE COMPANY KNOW WHEN I ENCOUNTER PROBLEMS IN USING THE APP.

ADVOCACY

I WILL GIVE THE FITNESS APP A HIGH RATING ON THE APP STORE.

I WILL RECOMMEND THE FITNESS APP TO OTHERS.

I WILL RECOMMEND THE FITNESS APP TO MY FRIENDS AND RELATIVES.

HELPING

I HELP OTHER APP USERS IN NEED.

I HELP OTHER APP USERS IN USING THE APP.

I HELP OTHER APP USERS USE THE APP CORRECTLY.

I GIVE ADVICE TO OTHER APP USERS.

TOLERANCE

I STILL ACCEPT THE APP EVEN IF IT DOES NOT WORK AS EXPECTED.

I WORK AROUND FAILURES IN THE FITNESS APP.

I WAIT PATIENTLY EVEN IF THE APP TAKES A LONG TIME TO LOAD.

The second part : Personal basic information

1.Your gender ?

Men and women

2.Your age ?

Under 18 years old 18-25 years old 26-35 years old 36-45 years old 45 years old and above

3.Your education level ?

High school and below junior college undergraduate master and above

4.Your monthly income ?

3000 yuan and below 3001-5000 yuan 5001-8000 yuan and above 8000 yuan

5.What is the average number of times you use fitness apps per month ?

3 times or less 4-8 times 9-15 times 15 times or more
