# Supplementary material for: Relationship between customer knowledge management and the value co-creation of fitness application customers: Mediating role of flow experience
Source: PLoS One. 2024 Oct 29;19(10):e0311988. doi: 10.1371/journal.pone.0311988 (PMC11521290; doi:10.1371/journal.pone.0311988)
Supplement: Attachment — Submitted filename: Revision of Manuscript response.docx [file pone.0311988.s003.docx]

Revision of Manuscript PONE-D-24-07863

Submitted to PLos One

**Relationship between Customer Knowledge Management and the Value Co-Creation of Fitness Application Customers: Mediating Role of Flow Experience**

Chao Wang^1^ , Zhigang Wang^2^ , Liandi Liu^3^ and Kai Hua^1*^

^1^Wuhan Sports University, Football College, Wuhan Hubei, China 430079.

^2^Wuhan Sports University, Economics and Management College, Wuhan Hubei, China 430079.

^3^ Renmin Hospital, Hubei University of Medicine, Shiyan, China 442000.

*corresponding author

First of all, we would like to express our sincere gratitude for the acknowledgment and acceptance of our manuscript by the reviewers and editors. We deeply appreciate your recognition of our work. In the remainder of this response, we explain point-by-point on how the issues raised by the reviewers were addressed in the revised version of the manuscript. The changes have been highlighted in the resubmitted manuscript.

**Response to the editor**

**Comment:** The paper investigates CKM, flow, and value co-creation in fitness apps. While well-organized, it's overly lengthy. It lacks clarity on why only fitness apps are studied and how flow is induced. Empirical reporting needs clarification on participant scenarios and temporal sequencing. Writing inconsistencies and grammatical errors persist, diminishing its publication suitability. Consequently, regretfully, the paper cannot be accepted for publication in its present state.

**Response:** Dear editor, thank you very much for your comments. According to your suggestions, the newly revised manuscript has made the following revisions :

1. The newly revised manuscript shortens the length.
2. About the reason why the fitness APP was studied, I made a demonstration in the introduction, 2.1 and 2.2 chapters of the manuscript.

“Introduction-As a product that integrates the social media interaction and sports fitness, fitness apps have become an important platform for customer value creation. Through diverse interaction channels, such as virtual brand communities, live interactions, and sports communities, customers can establish social contact with enterprises or other customers, and engage in innovation activities(e.g., provide Feedback and Experience on products), thereby facilitating the development of fitness apps. The present study aimed to uncover this mechanism in the context of fitness apps. Moreover, studies have focused on knowledge management or flow experience but have not investigated their effects on customer value co-creation. In particular, customer knowledge management aids in value co-creation by improving customers’ flow experience. Therefore, this study proposed and tested a model based on customer-dominant (C-D) logic, in which customer knowledge management affects customer value co-creation through customer flow experience in fitness apps.

2.1-In C-D logic, customers are the main source of value^[14]^. Moreover, customer value co-creation helps businesses to gain a more effective understanding of customers and to meet the heterogeneous needs of their customers (Figure 1)^[21]^. In the internet platform and virtual brand community, through interactive, businesses can apply artificial intelligence and data analysis to obtain customer insights, or fitness online classes can be provided to meet the needs of specific groups of customers according to their level and goals.^[22]^ For example, through the popular Chinese fitness app Keep, 21,200 online fitness classes were provided to 1.3 million users in December 2022. In particular, live fitness classes enable direct interactions between users and the coach, for example, for the adjustment of the course content according to the user’s needs. By 2022, Keep provided 9100 live online fitness classes to 17.1 million users. Therefore, understanding the crucial role of customer value co-creation and participatory process of customer value co-creation in the context of fitness apps is the major priority of fitness industry.

2.2-A fitness app is a product involving online human–computer interaction; in such products, customer flow experience is simple to generate and has a crucial impact on customer behaviors. Furthermore, customers can create value by sharing their experiences(like ideas, information, problems, needs etc.) in virtual sports communities or on fitness platforms^[14-15]^, Customer knowledge management has a positive effect on customer value co-creation. However, no study has investigated customer value co-creation in the context of fitness apps. Studies have investigated the outcomes of customer value co-creation in sports^[26-28]^, such as customer satisfaction or loyalty. Given that they facilitate social media and multiagent interactions, fitness apps have transformed the generation of such co-creation. Thus, the present study explored the role of customer value co-creation in the development of fitness apps.

In brief, to compensate for the shortcomings of previous research, the present study considered variables such as customer knowledge management and flow experience when exploring the influencing factors and participatory process of customer value co-creation in the context of fitness apps; the objectives were to enrich the existing literature regarding customer value co-creation in sporting contexts and to provide a novel research perspective to aid in the development of fitness apps.”

3. About the reason why the variable of flow experience is included in the study, I cited the case in the introduction part and the value co-creation theory part of the manuscript.

“Introduction-In fitness, flow experience is key to customer experience, and flow can be affected by the form of participation and the type of media used. Customers have a better perception of and participate more actively in fitness apps that help them attain the flow state more easily during exercise, thus aiding in value co-creation. Various exercises, such as dancing, cycling, and yoga, can be integrated on apps to help customers achieve flow and have a more favorable fitness experience overall. Attaining such flow leads customers to be more relationally and emotionally invested in the app and its community; thus, they are more likely to share their fitness journey and achievements with other users online.

**2.1 Value co-creation theory-**According to value co-creation theory in the context of C-D logic, customer knowledge management can be used to improve value co-creation by enhancing flow experience in fitness apps. This is exemplified by Keep. On this app, health consultants recommend exercise levels, fitness goals, everyday exercises, and dietary options tailored to users, enabling users to more effectively immerse themselves in the online fitness classes provided. The flow state then causes this user to engage with the app via a social feature where he recommends the specific exercise experience to other users. The Keep app leverages the knowledge shared by customers among themselves or with the company (i.e., customer knowledge management) for value co-creation, thus providing products and services that help customers achieve the flow state. This approach has resulted in 36.4 million active users and approximately 3.62 million subscribers monthly on the Keep app, with a membership penetration rate of approximately 10%. Thus, customer knowledge management, flow experience and value co-creation in C-D logic were considered in this study.”

4. In the empirical research section of the third chapter of the manuscript, the interviewees participation situation and the time of participation in the survey have been corrected.

“**3.1 Study Sample**-Fitness apps in China are utilized for maintaining health records (e.g., Gudong and Yue Running Circle), or they provide assistance with maintaining or improving fitness (e.g., Keep, Millet Sports, and BooHee Health). These apps also provide consultations regarding fitness or diet or are subscription services. This study focused on fitness apps applied for maintaining health records and providing fitness assistance because they feature frequent customer–customer and customer–business interactions and they help customers achieve their goals and engage in co-creation. To ensure the authenticity and reliability of the research sample, we introduced a screening question at the outset of the questionnaire specifically designed to identify these users.

**3.3 Pilot Study-**To refine the questionnaire, a pilot study involving 30 respondents was conducted from October 1 to 15, 2021. All participants provided their written informed consent, and the data were anonymized and used only in the context of the present study. We only included respondents who used apps for maintaining health records and providing fitness assistance.

3.4 Research Implementation-The formal survey was administered on the Questionnaire Star platform from October 17, 2021, to June 1, 2022. Convenience sampling was adopted to recruit the study sample. Respondents who completed the questionnaire were from college classes or fitness groups. Most respondents were from Fujian, Hubei, or Henan Province.”

5.The problem of consistent writing of ' APP ' in the manuscript has been corrected. And the grammatical errors in the manuscript have been corrected.

**Response to the 1st Reviewer**

**Comment:**The paper studies the effect of customer knowledge management and flow experience on customer value co-creation and the mediating role of flow experience in the context of fitness apps. The authors collected data using survey and conducted structural equation modeling (SEM) to test the hypotheses. The topic of the paper is very interesting. Here are my suggestions:

1. H1 proposes a positive effect. However, H1a and H1b do not have a direction. I would recommend add a direction to H1a and H1b hypotheses. The same logic applies H2.

2. Common method bias. The paper does not address the common method bias. I would recommend address the issue using statistical test.

**Response:** Dear reviewer 1, Thank you very much for reviewing the manuscript. According to your review of the manuscript, I made the following modifications in the revised manuscript :

1.The relationship or direction of the influence between the hypotheses has been modified.

“H1a: Customer knowledge management significantly and positively affects customer perceived enjoyment.

H1b: Customer knowledge management significantly and positively affects customer attention focus.

H2a: Perceived enjoyment significantly and positively affects customer participation behavior.

H2b: Perceived enjoyment significantly and positively affects customer citizenship behavior.

H2c: Attention focus significantly and positively affects customer participation behavior.

H2d: Attention focus significantly and positively affects customer citizenship behavior.”

2.The common method bias test has been supplemented in the fourth chapter of the manuscript.

“4.2 Common Method Bias-In this study, procedural control was used to minimize common method bias given that all data were collected using a single questionnaire, and statistical control was then applied to test for common method bias.

Specifically, in this study, the surveys were anonymized, the order of questions disrupted, and guides were added to highlight key content for reducing common method bias. No major common method bias was detected; a Harman’s single factor test revealed a common factor interpretation rate, which was less than the requisite maximum of 40%.”

**Response to the 2st Reviewer**

**Comment:**This paper includes a study examining the relationship between customer knowledge management (CKM), flow, and value co-creation within the domain of fitness applications. The paper studies an interesting topic, using a set of well-established constructs from the literature. I enjoyed reading the paper and learning about the topics. However, there is a vast area for improvement for the paper as it currently stands. I’ll focus my review on some of the critiques and how they could potentially be addressed.

**Response:** Dear reviewer 2, thank you very much for your review and recognition of the manuscript. Thank you very much for the review proposal. Next, I will modify and reply one by one according to your suggestions. Thank you.

Streamline the Introduction and Theory sections
The main critique I have is that the paper is far too long. There is only one study, yet the paper is around 35 pages. The organization by section is nice on the one hand. On the other hand, it is unclear to the reader why there needs to be an introduction, theoretical background, and hypothesis development sections. At the very least, I’d suggest combining the theoretical background and hypothesis development sections.

**Response:** The introduction and theoretical basis have been deleted. Specifically, the introduction deletes some meaningless paragraphs and optimizes the language of some paragraphs. The theoretical basis part combines the theoretical background and the hypothesis part.

**“1 Introduction**

With increasing affluence and given the impact of the COVID-19 pandemic, Chinese people have become increasingly health conscious; thus, fitness apps have proliferated to meet these health needs. According to QuestMobile, the number of downloads of fitness apps, such as Keep, Nike+, Daily Yoga, and Pleasure Circle, has increased sharply. As of February 2021, 780 million users utilized “cloud sports” technology on fitness apps, and nearly 60 million people used vertical fitness apps. Thus, China’s mobile online fitness industry has high market potential, and the development of fitness apps that fulfill specific fitness needs of the Chinese population in the market is a major priority for app enterprises.

...

The contribution of this study is twofold. First, it extends value co-creation theory to the context of fitness apps; the results indicate the importance of value co-creation for the fitness apps of enterprises. Second, by demonstrating how these three factors are interrelated in the context of fitness apps, it bridges the literature gap on customer knowledge management, flow experience, and customer value co-creation.”

**2 Literature review**

**2.1 Value co-creation theory**

Value co-creation theory has drawn considerable academic attention. This theory has evolved from being based on good-dominant (G-D) logic, where value is created only by the goods provided by businesses to customers; to service-dominant (S-D) logic, where customer services are considered the main contributor to value and are the foundation of economic exchange and which emphasizes high levels of business–customer interactions than those in G-D logic; to the current iteration of C-D logic, where value is derived from value co-creation, a flexible value network, and personalization of the goods and services provided.

...

Thus, customer knowledge management, flow experience and value co-creation in C-D logic were considered in this study.

**2.2 Customer Value co-creation in Fitness Apps**

Studies have categorized customer value co-creation into co-creation resulting from customer participation and co-creation resulting from customer citizenship, both of which are key in fitness apps (Table 1).

...

In brief, to compensate for the shortcomings of previous research, the present study considered variables such as customer knowledge management and flow experience when exploring the influencing factors and participatory process of customer value co-creation in the context of fitness apps; the objectives were to enrich the existing literature regarding customer value co-creation in sporting contexts and to provide a novel research perspective to aid in the development of fitness apps.

**2.3 Theoretical and Hypothesis Development**

...

H1a and H1b pertained to the relationships of customer knowledge management with the perceived enjoyment and attention focus dimensions of flow experience, respectively. H2a, H2b, H2c, and H2d pertained to the relationships of the aforementioned two dimensions of flow experience with the participation and citizenship dimensions of customer value co-creation. Finally, H3a and H3b pertained to the mediating effects of flow experience on the relationship between customer knowledge management and customer value co-creation.”

Improve the Concreteness of the Idea
For the background/introduction of the paper, it is a bit too general as it currently reads. Even after finishing the paper, I was left wondering why the study examines only fitness apps. Why (or why not) would the theoretical chain between CKM, Flow, and Co-Creation only exist for fitness apps? The authors note that fitness apps are popular in China and globally, which I agree with. But the decision to exclusively center the paper on the mobile fitness industry could be defended stronger. It is also worth noting (you mention this in section 4.3) that there are thousands of different fitness apps, meaning that there are endless different specific features, user interfaces, and recommendation systems that will vary depending on which specific app a participant is thinking about in your study. Value co-creation is also a large umbrella term. Why not just study rating of the app or word-of-mouth intentions?

**Response:** In the manuscript, I mainly revised the two parts of theory and practice on why to study the concept of fitness app. First of all, in the theoretical part, through combing the influence relationship of each variable in the introduction, it points out the research questions in the study of fitness app customer value co-creation. At the same time, the literature review emphasizes this research gap and highlights the importance of studying fitness app customer value co-creation.

**“1 Introduction-** As a product that integrates the social media interaction and sports fitness, fitness apps have become an important platform for customer value creation. Through diverse interaction channels, such as virtual brand communities, live interactions, and sports communities, customers can establish social contact with enterprises or other customers, and engage in innovation activities(e.g., provide Feedback and Experience on products), thereby facilitating the development of fitness apps . The present study aimed to uncover this mechanism in the context of fitness apps. Moreover, studies have focused on knowledge management or flow experience but have not investigated their effects on customer value co-creation. In particular, customer knowledge management aids in value co-creation by improving customers’ flow experience. Therefore, this study proposed and tested a model based on customer-dominant (C-D) logic, in which customer knowledge management affects customer value co-creation through customer flow experience in fitness apps.

The following research questions were also explored:

RQ1. How are customer knowledge management, flow experience, and customer value co-creation interrelated in the context of fitness apps?

RQ2. Does flow experience mediate the relationship between customer knowledge management and customer value co-creation in the context of fitness apps?

**...**

**2Literature review**

**...**

A fitness app is a product involving online human–computer interaction; in such products, customer flow experience is simple to generate and has a crucial impact on customer behaviors. Furthermore, customers can create value by sharing their experiences(like ideas, information, problems, needs etc.) in virtual sports communities or on fitness platforms, Customer knowledge management has a positive effect on customer value co-creation. However, no study has investigated customer value co-creation in the context of fitness apps. Studies have investigated the outcomes of customer value co-creation in sports, such as customer satisfaction or loyalty. Given that they facilitate social media and multiagent interactions, fitness apps have transformed the generation of such co-creation. Thus, the present study explored the role of customer value co-creation in the development of fitness apps.

...”

Secondly, in the part of practical demonstration, in the relevant theoretical basis, by referring to the case and application data of keep and combining with the theoretical basis, the manuscript once again demonstrates why the customer value co-creation of fitness app is explored. At the same time, based on the value co-creation theory of customer-led logic and related case data, it also demonstrates the relationship between knowledge management and flow experience and customer value co-creation. Furthermore, it improves the concreteness of the concept in the manuscript.

“**2.1 Value co-creation theory**

Value co-creation theory has drawn considerable academic attention. This theory has evolved from being based on good-dominant (G-D) logic, where value is created only by the goods provided by businesses to customers; to service-dominant (S-D) logic, where customer services are considered the main contributor to value and are the foundation of economic exchange and which emphasizes high levels of business–customer interactions than those in G-D logic; to the current iteration of C-D logic, where value is derived from value co-creation, a flexible value network, and personalization of the goods and services provided.

In C-D logic, customers are the main source of value. Moreover, customer value co-creation helps businesses to gain a more effective understanding of customers and to meet the heterogeneous needs of their customers (Figure 1). In the internet platform and virtual brand community, through interactive, businesses can apply artificial intelligence and data analysis to obtain customer insights, or fitness online classes can be provided to meet the needs of specific groups of customers according to their level and goals. For example, through the popular Chinese fitness app Keep, 21,200 online fitness classes were provided to 1.3 million users in December 2022. In particular, live fitness classes enable direct interactions between users and the coach, for example, for the adjustment of the course content according to the user’s needs. By 2022, Keep provided 9100 live online fitness classes to 17.1 million users. Therefore, understanding the crucial role of customer value co-creation and participatory process of customer value co-creation in the context of fitness apps is is the major priority of fitness industry.

According to value co-creation theory in the context of C-D logic, customer knowledge management can be used to improve value co-creation by enhancing flow experience in fitness apps. This is exemplified by Keep. On this app, health consultants recommend exercise levels, fitness goals, everyday exercises, and dietary options tailored to users, enabling users to more effectively immerse themselves in the online fitness classes provided. The flow state then causes this user to engage with the app via a social feature where he recommends the specific exercise experience to other users. The Keep app leverages the knowledge shared by customers among themselves or with the company (i.e., customer knowledge management) for value co-creation, thus providing products and services that help customers achieve the flow state. This approach has resulted in 36.4 million active users and approximately 3.62 million subscribers monthly on the Keep app, with a membership penetration rate of approximately 10%. Thus, customer knowledge management, flow experience and value co-creation in C-D logic were considered in this study.”

Another question I had while reading the paper was how exactly flow is being induced. The key theoretical concepts are explained (although I would suggest moving the explanations to be earlier on in the paper), yet I had a tough time understanding exactly what is going through a consumer’s mind and in their behavior. Providing a specific example on the first page describing exactly what features of CKM can induce a flow state in a customer, which then leads this customer to co-creation would be extremely beneficial in my opinion. For example, I did not understand how a flow state is being elicited (is the customer experiencing a flow state from using the app itself, such as logging a run OR is the flow state induced from the exercise itself, such as going for the run). If you could give an example along the lines of, “the Nike training app suggests a 3 mile run to the user based on his data. The user then completes the run and experiences a flow state while doing so. The flow state then causes this user to engage with the app via a social feature where he recommends the specific run route to other users in his area”. I’m not sure if this is how you picture the effect occurring in real life, but if so it would be beneficial to spell out.

**Response:** The reason why the flow experience was introduced has been clarified in the manuscript. At the same time, some practical cases are cited on the introduction and the literature review section.

“**1 Introduction**

Under customer-led logic, value co-creation is a dynamic and social process that is based on company–stakeholder interactions, and it can promote mutual trust and communication between the company and its stakeholders. In particular, the experience, knowledge, and ideas shared by customers on online platforms can be leveraged by enterprises to more effectively serve meet customer needs. Thus, customer experience is central to the perceptions and subsequent actions of customers, especially in value co-creation.

In fitness, flow experience is key to customer experience, and flow can be affected by the form of participation and the type of media used. Customers have a better perception of and participate more actively in fitness apps that help them attain the flow state more easily during exercise, thus aiding in value co-creation. Various exercises, such as dancing, cycling, and yoga, can be integrated on apps to help customers achieve flow and have a more favorable fitness experience overall. Attaining such flow leads customers to be more relationally and emotionally invested in the app and its community; thus, they are more likely to share their fitness journey and achievements with other users online.

**2.1 Value co-creation theory**

According to value co-creation theory in the context of C-D logic, customer knowledge management can be used to improve value co-creation by enhancing flow experience in fitness apps. This is exemplified by Keep. On this app, health consultants recommend exercise levels, fitness goals, everyday exercises, and dietary options tailored to users, enabling users to more effectively immerse themselves in the online fitness classes provided. The flow state then causes this user to engage with the app via a social feature where he recommends the specific exercise experience to other users. The Keep app leverages the knowledge shared by customers among themselves or with the company (i.e., customer knowledge management) for value co-creation, thus providing products and services that help customers achieve the flow state. This approach has resulted in 36.4 million active users and approximately 3.62 million subscribers monthly on the Keep app, with a membership penetration rate of approximately 10%. Thus, customer knowledge management, flow experience and value co-creation in C-D logic were considered in this study”.

Empirical Model and Study Reporting
Figure 2 is helpful in outlining exactly what your hypotheses are and how everything is connected in your conceptual model. The convenience sampling method used is not ideal, especially since the ample appears to be almost entirely 18-25 year olds who use fitness apps. If available, why not use an online participant recruitment platform like Prolific or Amazon Mechanical Turk? Table 2 is helpful, but I would suggest spending more time explaining the backdrop of the study. The questions/concepts are somewhat clear, but I was unsure what specific scenario or fitness app your participants were thinking about/responding to when answering these questions. Did you ask participants to download a specific app? Or are these questions in reference to an app they’ve already downloaded? Or are you just asking the questions more abstractly?

**Response:** Dear reviewers, it is necessary to explain to you that the age distribution of the survey samples in the study is in line with the demographic characteristics of the Chinese fitness application users, so the convenience sampling is reasonable.

“**3.4 Research Implementation**

Most respondents (93.3%) were aged 18 to 25 years, followed by the age group of 26 to 35 years (3.7%). This distribution was reflective of the age distribution of the fitness app–using population in China, as reported in the China Mobile Fitness Industry Report released by iResearch Consulting in 2021”.

Considering the factors such as the survey object and the research area, the research uses the most authoritative online survey platform in China, so the platform of Prolific or Amazon Mechanical Turk is not considered. Hope to get your understanding. Thank you.

Table 2 in the manuscript summarizes the source of the relevant variables and the specific division of the variables. Therefore, in order to better explain the background of its research, we add some original reference variable items in Table 2. Please review it again. Thank you.

In the research sample chapter, the revised manuscript has clarified the research scenario in which the subjects participate, that is, the type of fitness app used. At the same time, in the questionnaire, we did not ask subjects to download specific apps. The setting of the question is based on the subject 's use experience, and the relevant test items are also set up in the questionnaire to identify whether the subject has experience. Only experienced subjects will participate in the survey.

“**3.1 Study Sample**

Fitness apps in China are utilized for maintaining health records (e.g., Gudong and Yue Running Circle), or they provide assistance with maintaining or improving fitness (e.g., Keep, Millet Sports, and BooHee Health). These apps also provide consultations regarding fitness or diet or are subscription services. This study focused on fitness apps applied for maintaining health records and providing fitness assistance because they feature frequent customer–customer and customer–business interactions and they help customers achieve their goals and engage in co-creation. To ensure the authenticity and reliability of the research sample, we introduced a screening question at the outset of the questionnaire specifically designed to identify these users”.

Again, I like the model you outline and report in Figure 3. The question I have here is whether you can explain why flow comes before participation behavior. This point may not be valid if you had explained the study design better, but since I am confused I’ll ask if it is possible for flow experience to happen simultaneously during customer value co-creation. I’m skeptical you can claim mediation here since what you have is not actually an experiment with a manipulated independent variable preceding the dependent variable. I’d suggest an experiment where you experimentally manipulate CKM, then measure flow/attention, and then measure value co-creation.

**Response:** Dear reviewers, based on literature review and theoretical logic, I have clarified the theoretical connection between flow experience and value co-creation behavior in the second chapter, and explained the research design. Therefore, there is no need to add other experiments in this manuscript. In addition, thank you very much for the research idea of manipulating CKM to verify the relationship between flow experience and value co-creation. At present, I have further carried out relevant research according to your ideas. Thank you very much for your help, thank you.

Improvements in Writing
There are also many instances of typos/inconsistencies/grammatical errors in the manuscript which made it difficult to interpret. The word “APP” is arbitrarily capitalized in some cases but not others. There are hanging spaces in some areas whereas others need a space after a period. A few sentences were not complete. It could also be important to create a stronger hook for the importance of the finding. I’d imagine all fitness app designers strive to delight their customers, leading to enjoyment of their app and thus wanting to co-create. However, what could a designer specifically take away from the survey to specifically enhance their app?

**Response:** Dear reviewer, Thank you for your comments . I have unified the writing of the word app in the manuscript, and I have also modified the applicability of the hanging space in the manuscript. For some grammatical errors, I also made corresponding corrections. Finally, with regard to the improvement measures for designers ( enterprises ), I have indicated in the 5.1.1 Chapter of the manuscript. Please review it again, thank you.

“**5.1.1 Practical implications**

Customer knowledge management is key for fitness app enterprises. The insights obtained through customer knowledge management can be used to enhance customer flow experience, thus promoting customer value co-creation for app development.

First, fitness apps must improve customer knowledge management approaches to promote value co-creation. The content generated through co-creation provides a large amount of data; such data can be leveraged through customer knowledge management to provide training programs tailored to users. In particular, integrating instant feedback and reward functions into fitness apps should be prioritized to promote user interactions.

Second, fitness apps must promote the customer flow experience to strengthen the psychological connection of customers to the enterprise. In the experience economy era, fitness app users can choose products that match their personal preferences. Therefore, to improve customers’ perception of fitness products and services, enterprises can mine data on customer characteristics to create exclusive, customized, or modifiable fitness products and services. Enterprises can also expand service scenarios and improve product distribution or after-sales services to enhance customer experience.

Third, fitness apps can promote customer value co-creation and strengthen their relationship with customers. For this purpose, enterprises must continue to innovate in how they connect with customers and must obtain insights of the target market to effectively generate customer value co-creation. Innovative interactive communication could replace traditional user interaction in virtual brand communities and live interactions and customer functions, and permissions can be expanded to promote customer browsing, participation, and feedback reporting. Furthermore, reward mechanisms can be integrated into user interfaces to stimulate co-creation..”

Finally, we would like to express our great appreciation to the reviewers for your comments on our paper. We believe that these improvements will help improve the quality and readability of the article, and hope that this latest version meets your requirements and expectations.

Best regards，

Chao Wang
